# Voice Analysis in Dogs with Deep Learning: Development of a Fully Automatic Voice Analysis System for Bioacoustics Studies

**DOI:** 10.3390/s24247978

**Published:** 2024-12-13

**Authors:** Mahmut Karaaslan, Bahaeddin Turkoglu, Ersin Kaya, Tunc Asuroglu

**Affiliations:** 1 Department of Computer Engineering, Konya Technical University, 42250 Konya, Turkey; mkaraaslan@ktun.edu.tr (M.K.); ekaya@ktun.edu.tr (E.K.); 2 Department of Artificial Intelligence and Data Engineering, Ankara University, 06830 Ankara, Turkey; turkoglub@ankara.edu.tr; 3 Faculty of Medicine and Health Technology, Tampere University, 33720 Tampere, Finland; 4 VTT Technical Research Centre of Finland, 33101 Tampere, Finland

**Keywords:** automatic behavior analysis, bioacoustics, CNN, audio processing

## Abstract

Extracting behavioral information from animal sounds has long been a focus of research in bioacoustics, as sound-derived data are crucial for understanding animal behavior and environmental interactions. Traditional methods, which involve manual review of extensive recordings, pose significant challenges. This study proposes an automated system for detecting and classifying animal vocalizations, enhancing efficiency in behavior analysis. The system uses a preprocessing step to segment relevant sound regions from audio recordings, followed by feature extraction using Short-Time Fourier Transform (STFT), Mel-frequency cepstral coefficients (MFCCs), and linear-frequency cepstral coefficients (LFCCs). These features are input into convolutional neural network (CNN) classifiers to evaluate performance. Experimental results demonstrate the effectiveness of different CNN models and feature extraction methods, with AlexNet, DenseNet, EfficientNet, ResNet50, and ResNet152 being evaluated. The system achieves high accuracy in classifying vocal behaviors, such as barking and howling in dogs, providing a robust tool for behavioral analysis. The study highlights the importance of automated systems in bioacoustics research and suggests future improvements using deep learning-based methods for enhanced classification performance.

## 1. Introduction

Extraction behavioral information from animal and human vocalizations has fascinated researchers for decades [1,2]. In the field of bioacoustics, sound-derived behavioral data are particularly valued for their critical role in understanding animal behavior and interactions with the environment [3]. These data offer critical insights into animal ecology, communication, and welfare.

Historically, animal behavior studies relied on visual or auditory recordings, requiring manual analysis, which was labor-intensive and time consuming [4]. Automated systems have since been developed to address the challenges [5].

Behavior analysis is crucial for understanding how animals interact with their environment. This study focuses on sound classification as a foundational step, enabling precise identification of vocal patterns and facilitating research in animal welfare, communication, and training. While we focused primarily on sound classification in this study, it is important to highlight that accurate classification of animal vocalizations serves as a foundational step for behavioral analysis. By enabling the precise identification of specific vocal patterns, our system provides a valuable tool for researchers in animal welfare, communication studies, and training applications. These insights can facilitate more nuanced investigations into behavioral dynamics, contributing significantly to bioacoustics research and its applications.

In bioacoustics, the primary challenge is not data collection but the labor-intensive review of long audio recordings [6]. Automated analysis of these recordings has substantial potential for advancing animal welfare by enabling more efficient monitoring of behavioral patterns [7]. Consequently, significant research efforts have been directed toward the development of automatic classifiers for detecting and classifying animal vocalizations [7,8,9].

The unique relationship between humans and dogs has fostered interspecies communication, with vocal signals conveying specific emotions such as stress, joy, or aggression [10]. Dogs express emotions through variations in acoustic parameters such as tonality, pitch, and inter-bark intervals [11]. These vocalizations provide valuable insights for veterinarians into behaviors such as warning signals, territoriality, aggression, celebration, pain, stress, and joy [12,13]. In some studies, the classification of dog vocalizations has even been employed for preliminary health diagnoses [14]. Yin and McCowan demonstrated that bark frequency and duration provide significant information for differentiating behaviors, such as distress or urgency [15]. Higher-pitched vocalizations typically reflect heightened emotional states, whereas lower pitches are indicative of calmer behaviors [11,16].

Yeo et al. developed an application for identifying individual dogs based on their vocalizations using a zero-cross-rate (ZCR) algorithm for detecting voiced regions and Mel-frequency cepstral coefficients (MFCCs) for feature extraction. Classification was conducted using the Dynamic Time Warping (DTW) algorithm, which, while effective, is constrained by its reliance on a limited database, reducing its applicability across diverse populations of dogs [17].

Tani and colleagues proposed an automated system for recognizing and classifying chewing activities in cows using a single-axis accelerometer and a sound recorder. They demonstrated that, while the accelerometer was effective for acoustic studies, classification accuracy was influenced significantly by environmental factors. Further improvement through deep learning techniques was suggested to enhance the robustness of the classification system [18].

Bishop et al. developed a multipurpose sound classifier for farm animals, including sheep, cattle, and dogs. The system utilized MFCCs, Discrete Wavelet Transform (DWT), and a Support Vector Machine (SVM) neural network, achieving promising results. However, the manual segmentation of sound clips posed a limitation, and the researchers emphasized the necessity of integrating automatic sound segmentation algorithms to increase practical applicability [19].

Nunes et al. focused on the classification of biting and chewing sounds in horses using MFCCs and a Long Short-Term Memory (LSTM) neural network. Their study revealed that noise in the data negatively impacted the system’s sensitivity, highlighting the need for further research into noise reduction techniques to improve accuracy [20].

Tsai and Huang developed a real-time Internet of Things (IoT) system for monitoring dogs’ emotional states using both audio and video inputs. They trained a region-based convolutional neural network (R-CNN) on spectrograms to classify emotional states, such as anger, sadness, and normal barking. This approach underscores the potential of integrating audio and video data for comprehensive real-time behavioral monitoring [21].

Convolutional neural networks (CNNs) have demonstrated remarkable success in image classification tasks and have been effectively adapted for sound processing using spectrograms as input features [22,23]. By leveraging transfer learning, researchers have successfully applied CNNs to audio classification, achieving notable results [24].

This study introduces a CNN-based system for detecting and classifying dog vocalizations. By leveraging spectral features obtained via STFT, MFCCs, and LFCCs, the system identifies barking and howling with high accuracy, providing both class and duration information for each segment. This represents a significant advancement in automated bioacoustics analysis. An initial preprocessing step was implemented to identify and segment relevant sound regions from audio recordings, utilizing sound energy information. The Short-Time Fourier Transform (STFT) was employed for feature extraction to preserve time-related information, producing Mel spectrograms as features. MFCC transformations were applied as an additional feature set due to their effectiveness in capturing meaningful sound characteristics. In addition, linear-frequency cepstral coefficients (LFCCs), which capture more detail in lower frequencies compared with MFCCs, were evaluated [25,26]. Spectrograms were subsequently transformed into one-dimensional images for classification.

Five different CNN architectures were employed for the classification task, with consistent training and testing splits, random state values, and epoch counts to ensure fairness in performance comparison. The evaluation included analyses of loss curves, confusion matrices, and performance metrics derived from these matrices.

Two classification systems were introduced, one capable of real-time (online) analysis and the other for offline processing, with the current focus on the offline system. The system processed audio files through segmentation and feature extraction, subsequently inputting these data into the classifier to generate behavioral outputs. This system represents a pioneering effort in the domain of fully automated sound analysis in animals, offering substantial potential for clinical research and bioacoustics.

The primary aim of this study was to develop an automated sound analysis system that detects voiced regions in raw audio recordings of dogs using the root mean square energy (RMSE) method, classifying these regions as either barking or howling through a CNN-based model. The CNN model was trained on spectral images obtained via STFT, allowing it to learn the characteristic spectral features of these vocalizations. The proposed system provides both class and duration information for each vocalization segment, representing a significant contribution to the automatic classification of dog vocalizations.

The continuation of the study is organized as follows. The Section 2, “Materials and Methods”, begins with a description of the dataset. The second part of the “Materials and Methods” section discusses the audio segmentation process as a preprocessing step. The third part covers the Fourier transforms used as feature extractors. The fourth part explains the classifier system. The fifth part introduces the proposed system that utilizes the methods described in the previous sections. In the Section 3, “Experimental Results and Discussion”, the CNN models used as classifiers in the proposed system are evaluated using various metrics. The Section 4, “Conclusions”, summarizes the outcomes of the study and provides suggestions for future research areas.

## 2. Materials and Methods (Background)

### 2.1. Dataset

A review of existing literature highlights a scarcity of datasets for canine vocalization classification. This issue is due to limited availability of publicly shared datasets and the inherent variability in canine vocal behavior. There is a clear need for more well-curated datasets to improve classification accuracy.

This study focuses on analyzing canine vocal behavior by detecting and classifying barking and howling using an open-source dataset. This approach aims to provide deeper insights into the context of these vocalizations.

The system was trained using the open-source “Audio Cats and Dogs” dataset, which includes 113 dog recordings and 164 cat recordings, totaling 598 min of dog sounds and 1323 min of cat sounds, all at a 16 kHz sampling rate. Dog sounds were categorized into 46 instances of barking and 57 of howling, with barking defined as sudden, repetitive sounds and howling as prolonged vocalizations.

The newly annotated dataset has been made publicly accessible to support validation and future research, addressing the lack of datasets for canine vocalization classification and promoting further research in this field.

### 2.2. Preprocessing (Audio Segmentation)

In bioacoustic or behavioral studies, sounds are collected as raw recordings. Detecting vocal segments from raw recordings is essential for training, testing, and analysis purposes. Accurate identification of these vocal segments ensures that only these segments are used for training and testing, and also facilitates their identification during the analysis process. For example, to accurately classify sounds such as barking and howling, these vocal segments must be detected and separated from the raw audio recordings. It is crucial that the model is trained specifically on these vocal segments and that the data used in the system focus on these segments. To achieve this, endpoint detection algorithms are employed to identify the vocal segments within the audio. Energy-based detection is the most commonly used method among these algorithms and has therefore been chosen for the proposed system.

In this approach, the energy of the input speech signal is calculated over a series of frames. A frame is considered a valid speech segment if its energy exceeds a predefined threshold; otherwise, the frame is classified as a silence segment. The root mean square energy (RMSE) method is used to detect the energy. Using this method, the energy of the audio signal is calculated using Equation (1) [27].

(1) $$RMSE=\sqrt{\frac{1}{k}\times \sum_{k=tK}^{\left(t+1\right)\times K-1}{s\left(k\right)}^{2}}$$

In this equation:

- RMSE: represents the root mean square energy, which indicates the average power of the sound signal.
- s(k): denotes the k−th sample of the sound signal. These samples represent the varying values of the signal over time.
- K: indicates the number of samples within a frame. This represents the small portions of the sound signal being analyzed.
- t: represents the index of the frames.

The equation computes the sum of squares of s(k) over a particular frame. This sum is then divided by K to compute the average. Finally, the square root of this average is taken to obtain the RMSE value. This process is used to calculate the average energy of the sound signal over a certain period of time. Typically, this value is used to determine the power or intensity of the sound signal.

As part of this study, the RMSE segmentation method is applied to sound recordings in isolated environments. In these environments, the energy of background noise is much lower compared with the energy of dog sounds. Therefore, the detection of vocal regions belonging to dogs is achieved with a simple manual energy threshold. This approach allows partial filtration of background noise. Furthermore, although it is assumed that high noise levels in isolated environments are primarily caused by the dogs, we prevent noise from other sources by examining the likelihood of the sound belonging to a specific class during the classification stage.

RMSE segmentation has proven successful in isolated environments within this study. However, in noisier environments, such as farms, further research is needed on noise reduction techniques.

**Figure 1:**
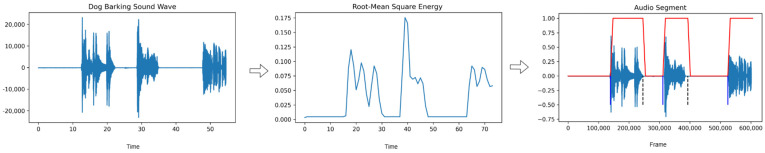
Sound segmentation process.

After applying the energy calculation process described in Equation (1) to the input sound, endpoints are determined. With these endpoints, the beginning and end of the voiced region are identified, and the desired region is extracted from the sound. This extracted segment is then fed into the trained model to achieve the desired outcome. The general flow of the sound segmentation process is illustrated in Figure 1.

### 2.3. Feature Extraction

For emotion analysis, researchers frequently recommend frequency-domain methods such as MFCCs [28]. Frequency-domain techniques have been widely utilized in the classification of animal emotions, with their superior performance well documented across numerous studies [29]. In particular, many works have demonstrated the efficacy of MFCCs and other frequency-domain feature extractors in the classification of dog vocalizations [21,30,31]. Accordingly, in this study, we employ frequency-domain features to enhance the accuracy of our analysis.

**Figure 2:**
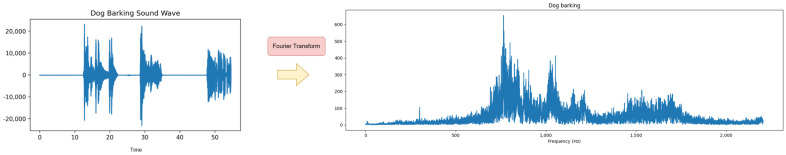
Fourier transform on audio data.

Frequency-based approaches offer distinct advantages for analyzing and interpreting sound signals. Specifically, the inherent complexities of sound signals offer limited information when examined solely in the time domain. To overcome these complexities and enable a more detailed analysis by decomposing the sound signals into their frequency components, the Fourier transform is widely used. The Fourier transform separates a signal into distinct frequency components [32]. In Figure 2, the time-domain sound waveform of a dog barking is shown, followed by its spectral distribution in the frequency domain after applying the Fourier transform.

**Figure 3:**
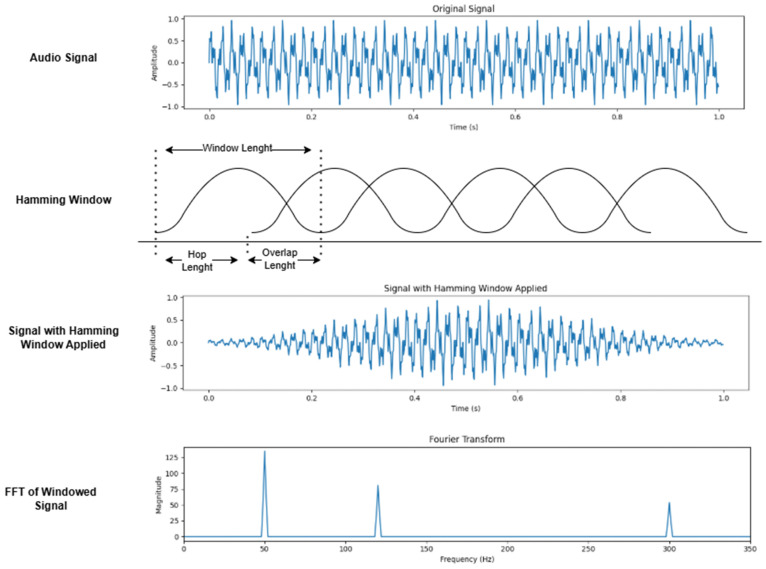
STFT conversion general system diagram [36].

When applying the Fourier transform to a sound signal, temporal information is lost, and the signal is transformed into an instantaneous representation of the entire waveform. Since real-time sound signals exhibit characteristics of time series, analyzing the changes over time is crucial. To address this issue, the Short-Time Fourier Transform (STFT) method is employed; in this approach, the sound signal is divided into frames at specified intervals, and Fourier transform is applied to each frame [33]. To mitigate the discontinuity between frames, a window function is applied to each frame, thereby preventing spectral leakage [34]. The Hamming window is a commonly used technique for this purpose [35]. Furthermore, to reduce data loss, overlapping is performed between frames, typically at ratios of ½ or ¼ of the frame length [36]. All stages of the STFT process are illustrated in Figure 3.

**Figure 4:**
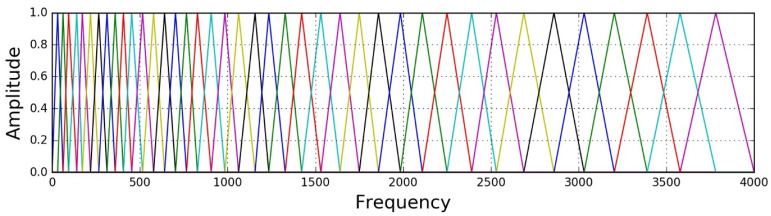
Triangle bandpass Mel filter bank.

Triangular filters model the nonlinear perception of frequencies by the human auditory system, making sound data meaningful [37]. Therefore, the use of Mel spectrograms provides a significant advantage in human perception-based labeling. The accuracy of human-generated labels necessitates that the model processes data in a manner akin to the human auditory system; otherwise, the likelihood of the model learning irrelevant information increases, potentially deviating from the intended purpose of the learning process. A typical Mel filter bank configuration with 128 triangular filters was used in our experiments, as illustrated in Figure 4.

The conversion to Mel frequency is carried out with the help of Equation (2).

(2) $$m=2595\log_{10}\left(1+\frac{f}{700}\right)$$

where f represents the frequency in Hertz (Hz). The Mel frequency scale, as defined in Equation (2), transforms linear frequencies to a scale that aligns more closely with human auditory perception. This approach is rooted in the foundational work by Stevens, Volkman, and Newman [38]. 

**Figure 5:**
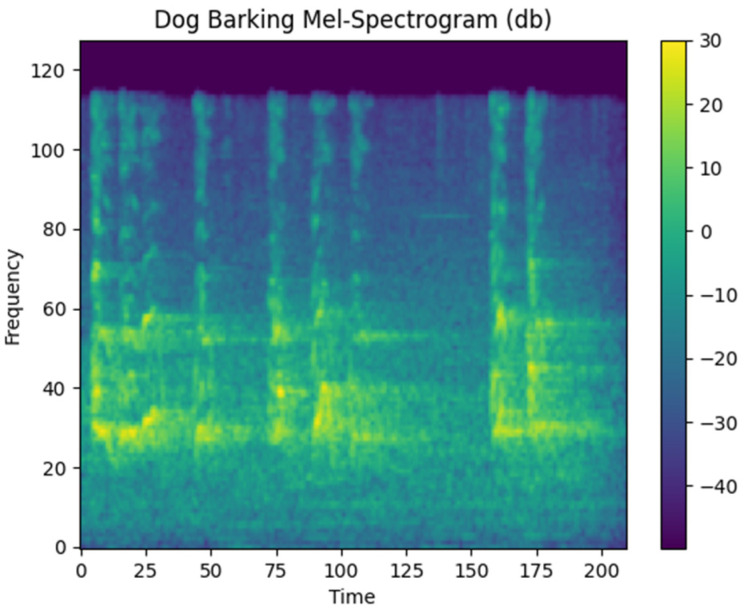
Mel-spectrogram output.

Mel spectrograms are recognized as effective features for deep learning-based sound algorithms. Therefore, in this study, one of the features we employ is the Mel spectrogram. An example Mel spectrogram output created in this study is shown in Figure 5.

**Figure 6:**
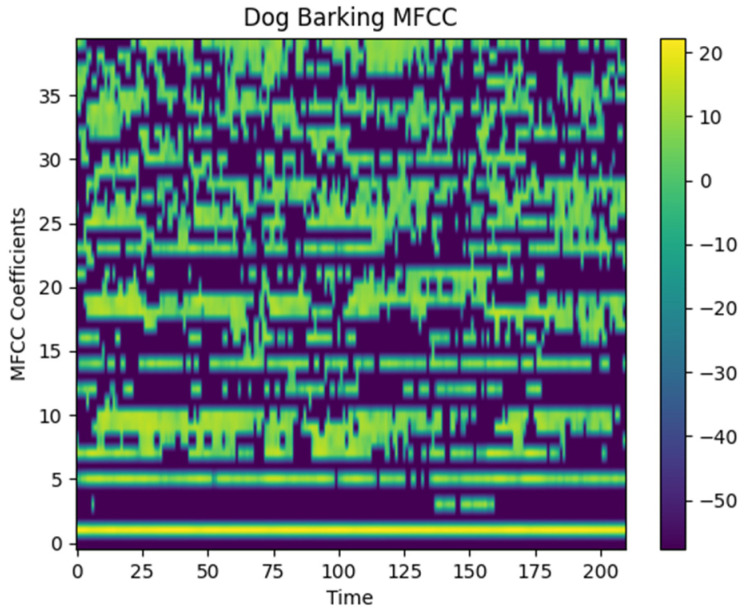
Sample MFCC output of the dataset.

Some studies in the literature have shown that high classification accuracy is achieved in voice classification with the MFCC transformation [39]. Researchers have successfully performed classification in livestock using the MFCC features in combination with CNN architectures [40]. MFCC features have also demonstrated high performance in emotion detection [41]. Motivated by the successful results reported in the literature, this study also evaluates the use of MFCCs for the classification of barking and howling emotions. For this purpose, the MFCC output of a sound in the dataset was created. The relevant MFCC output is shown in Figure 6.

**Figure 7:**
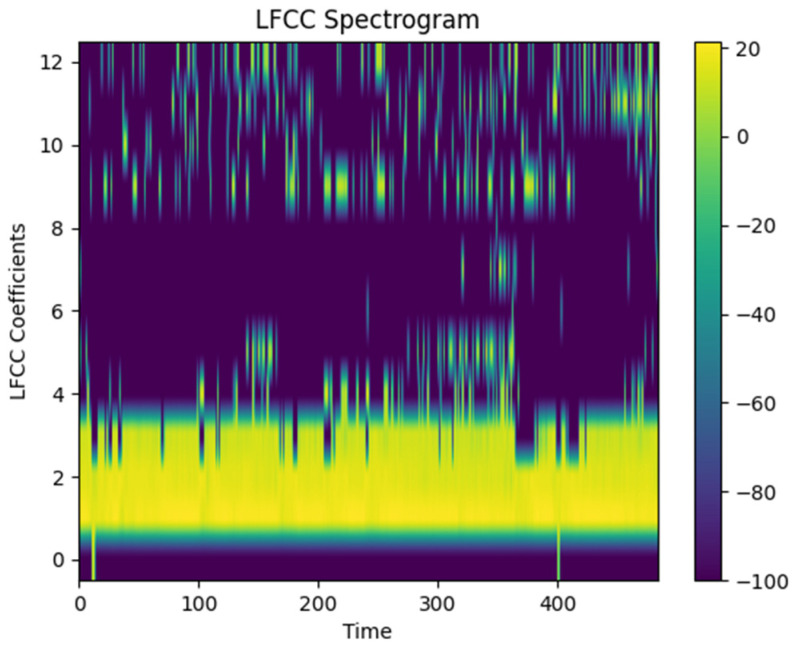
LFCC spectrogram.

LFCCs (linear-frequency cepstral coefficients) are a method used for extracting features from audio signals. They are commonly employed in areas such as speech recognition, sound classification, and audio signal analysis. LFCCs represent an audio signal linearly on the frequency axis and extract energy values from specific frequency bands by partitioning the signal accordingly. These energy values are subsequently processed through cepstral analysis, which aids in better understanding the spectral characteristics of the signal. The key difference between LFCCs and MFCCs (Mel-frequency cepstral coefficients) is that LFCCs operate on a linear frequency scale, while MFCCs utilize the Mel scale to represent frequencies in a manner more aligned with human auditory perception. Consequently, LFCCs can be particularly useful when detailed analysis of a wide frequency spectrum is required. The LFCC output obtained with sample data is shown in Figure 7.

### 2.4. Classification

**Figure 8:**
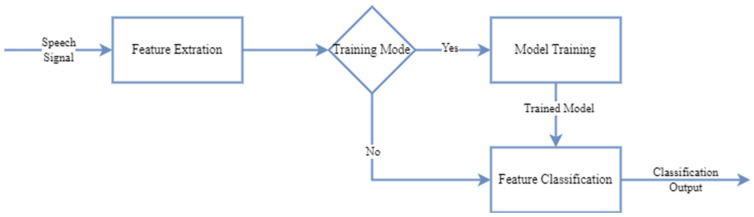
Block diagram of the speech recognition system [37,42].

For the classification process, a model is trained by applying feature extraction transformations on the labeled data in the dataset. The trained model is stored in a database for subsequent classification tasks. In the classification stage, the sound signal from which the feature is extracted is given to the model, and the class with the most appropriate match in the model in the database is presented as output. A block diagram of the system is shown in Figure 8 [37,42].

#### 2.4.1. Convolutional Neural Network (CNN)

Implementing a convolutional neural network (CNN) for speech recognition or speaker identification involves data collection, preprocessing, model design, and evaluation. A balanced dataset is created using public corpora like LibriSpeech or VoxCeleb, or through custom datasets. Preprocessing includes normalizing signals, resampling (e.g., 16 kHz), and noise reduction. Long recordings are segmented, and features are extracted using STFT, MFCCs, Mel spectrograms, and LFCCs.

The model architecture starts with an input layer for extracted features, followed by convolutional layers based on VGG or ResNet, using 3 × 3 or 5 × 5 filters with ‘same’ padding and ReLU activation. Max pooling reduces spatial dimensions, and the output is flattened and passed through fully connected layers with dropout to prevent overfitting. The final layer uses SoftMax for multi-class or sigmoid for binary classification.

The model is compiled with a suitable loss function (e.g., categorical cross-entropy) and an optimizer like Adam or SGD. Metrics such as accuracy, precision, recall, and F1-score are used for monitoring. The dataset is divided into training, validation, and test sets (70%, 15%, 15%). Training involves iterative updates, with validation after each epoch, and hyperparameter tuning through grid or random search.

Model evaluation uses metrics like accuracy, precision, recall, F1-score, and confusion matrix analysis. Learning curves are analyzed for convergence, and Grad-CAM visualizes model focus areas. The trained model is saved (e.g., HDF5) and deployed for real-time audio processing, with continuous monitoring to track performance.

Maintenance involves periodic re-training with new data to adapt to evolving patterns. A feedback loop collects data and user feedback for ongoing improvements, ensuring robust and adaptable performance.

#### 2.4.2. Classification Systems

**Figure 9:**
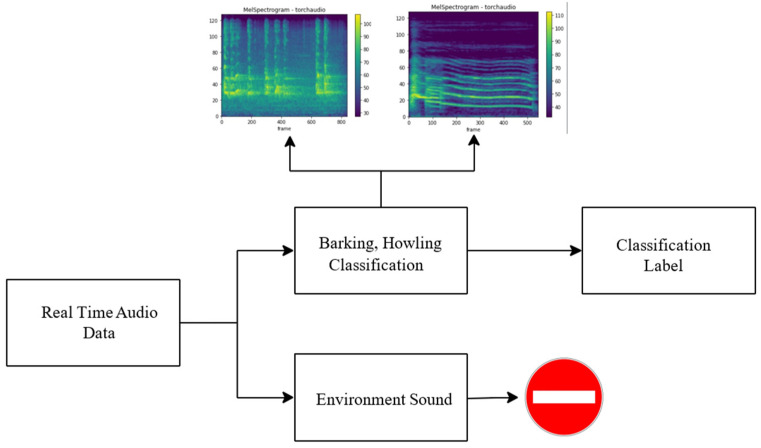
System design for Real-Time problems with different sounds.

For real-time audio analysis in environments with varying background sounds, the system design depicted in Figure 9 was developed. This system checks whether the incoming audio data correspond to the target classification source. If the audio source is intended for classification, the data are aggregated until the incoming data are no longer from the desired source. Once the data flow from the target audio source is complete, the system passes the aggregated audio to the classification model and generates the output.

**Figure 10:**
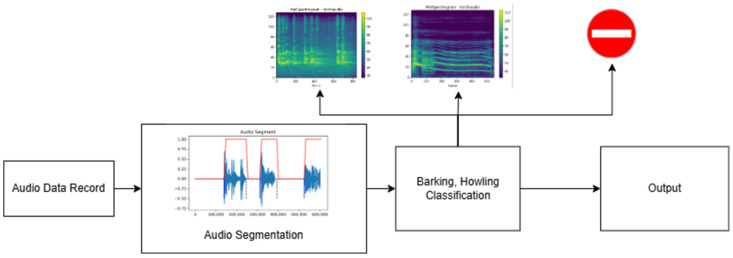
System design for offline and isolated environments.

In order to carry out the sound analysis process offline and in isolated environments, the system design shown in Figure 10 was made. In this system, the entire audio file given as input is segmented by the audio segmentation process and then subjected to classification.

Since the analysis problem to be used in the study needs to be performed offline and in isolated environments, improvements were made to the system shown in Figure 10.

#### 2.4.3. Model Training

The training was conducted on a Lenovo ThinkStation with an Intel Core i9, NVIDIA RTX A2000 GPU, and 64 GB RAM. PyTorch with CUDA was used to train and evaluate multiple models using both Mel spectrograms and MFCC features. Spectrograms were generated with a sampling rate of 22,050 Hz, hop length of 512, FFT size of 2048, and 128 Mel bands, with 40 MFCC coefficients, then resized to 256 × 256 pixels.

The dataset of 103 audio samples was split 80% for training and 20% for testing, using a consistent random state. Each model was trained with a batch size of 32 for 700 epochs.

#### 2.4.4. Classification Evaluations Metrics

Confusion matrices evaluate classification performance by calculating True Positives (TP), True Negatives (TN), False Positives (FP), and False Negatives (FN).

- TP: correctly classifies barking minutes.
- TN: correctly classifies howling minutes.
- FP: howling minutes classified as barking.
- FN: barking minutes classified as howling.

These metrics are calculated using the formulas given in Equations (3)–(8).

(3) $$Precision=\frac{TP}{TP+FP}$$

(4) $$Recall=\frac{TP}{TP+FN}$$

(5) $$F1Score=2\times \frac{Precision\times Recall}{Precision+Recall}$$

(6) $$Accuracy=\frac{TP+TN}{TP+TN+FP+FN}$$

From these values, key metrics are derived:

- Precision: proportion of correctly identified positive samples among predicted positives.
- Recall: proportion of actual positive samples correctly identified.
- F1-Score: harmonic mean of precision and recall.
- Accuracy: proportion of correctly classified instances overall.

For multi-class classification, Macro Average and Weighted Average are used:

- Macro Average: arithmetic mean of metric values (precision, recall, or F1-score) calculated independently for each class.
- Weighted Average: the average of metric values weighted by the number of samples (support) in each class.

(7) $$MacroAverage=\frac{1}{N}\sum_{i=1}^{N}{Metric}_{i}$$

(8) $$WeightedAverage=\frac{\sum_{i=1}^{N}\left({Metric}_{i}\times {Support}_{i}\right)}{\sum_{i=1}^{N}{Support}_{i}}$$

Here:

${Metric}_{i}$: represents the performance metric (e.g., precision, recall, or F1-score) for the i-th class.

${Support}_{i}$: refers to the number of samples in the i-th class.

N: the total number of classes.

### 2.5. Full Automatically Vocalization Analysis System

**Figure 11:**
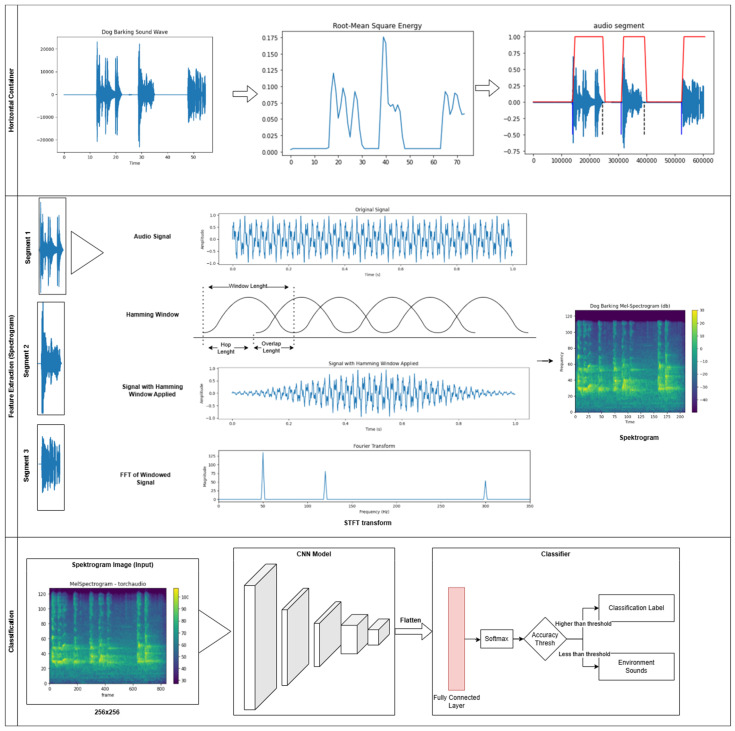
Fully automatic sound classification system.

Researchers first take voice recordings to conduct voice analysis in animals. As sound recordings are spread over time, the relevant sounds must first be extracted from the main audio file for classification. For this detection process, the audio segmentation algorithm described in the previous section is used. With this algorithm, audible regions in a recorded audio file are detected using the energy feature. The detected vocal regions are transformed for individual feature extraction. The segmented sounds from which feature extraction is made are given to the trained model and the classification output is obtained. If the accuracy of the classification exceeds the predefined threshold, the segments are labeled as barking or howling. Segments failing the accuracy threshold are labeled as environmental sounds. The design of the developed automatic system is given in Figure 11.

The figure illustrates that the fully automatic sound classification system takes a raw sound recording as input, segments it, extracts features, and classifies each segment. For each segment obtained during the segmentation phase, the class label, duration, average pitch value, and energy are calculated. Based on the classification results, the total duration and count are computed for each class. The obtained results are presented for interpretation by veterinarians.

**Figure 12:**
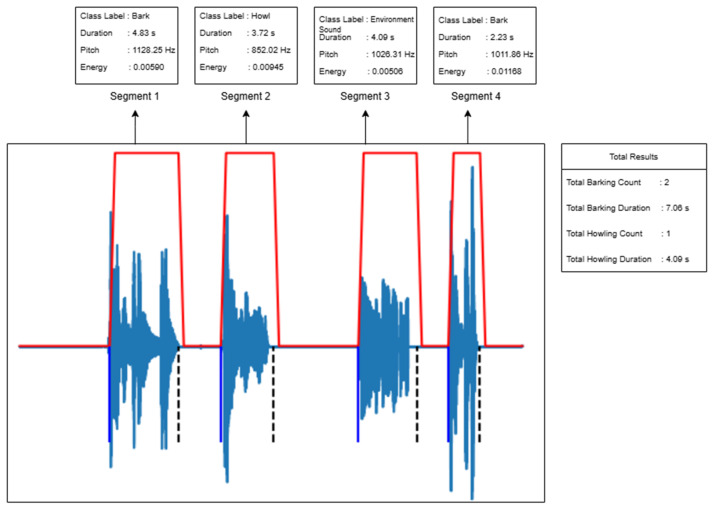
Sound classification system output.

The output obtained from a sample sound file using the fully automatic sound classification system is shown in Figure 12, providing an example of the system’s capabilities.

## 3. Experimental Results and Discussion

The experimental results presented in this section aim to demonstrate the effectiveness of the proposed automatic sound detection and classification system in accurately identifying and classifying dog vocalizations. These findings highlight the performance of various convolutional neural network (CNN) models and feature extraction methods, and compare them with traditional machine learning classifiers (including Support Vector Machines, K-Nearest Neighbors, Random Forest, and Naive Bayes). Table 1 summarizes the results obtained from these approaches, providing a comprehensive overview of model performances across different feature sets. This expanded comparison allows for a more thorough evaluation of the proposed deep learning approach, showcasing its advantages over conventional methods and further underscoring its potential to improve accuracy and reliability in the classification of dog vocalizations.

The results from the AlexNet model using Mel spectrograms indicate that the precision, recall, and F1-score for ‘Bark’ and ‘Howl’ are 0.83 and 0.78, respectively, with an overall accuracy of 0.81. While AlexNet can classify dog vocalizations reasonably well with Mel spectrograms, its performance is comparatively lower than other models. In contrast, using MFCC features yielded a precision, recall, and F1-score of 0.92 and 0.89 for ‘Bark’ and ‘Howl’, respectively, with an overall accuracy of 0.90. This suggests superior performance with MFCC features, emphasizing the impact of feature extraction on model efficacy. LFCC features resulted in a precision, recall, and F1-score of 0.90 for ‘Bark’ and 0.73 for ‘Howl’, with an overall accuracy of 0.81, indicating lower effectiveness compared with Mel spectrograms and MFCCs.

DenseNet, trained with Mel spectrograms, achieved a precision and recall of 0.92 and 0.89 for ‘Bark’ and ‘Howl’, respectively, resulting in an overall accuracy of 0.90. This demonstrates DenseNet’s high efficacy in distinguishing dog vocalizations using Mel spectrograms. When trained with MFCC features, the model achieved a precision and recall of 0.91 and 0.83, respectively, with an overall accuracy of 0.86, indicating a slight performance dependency on feature type. LFCC features yielded a precision of 0.91 for ‘Bark’ and 0.80 for ‘Howl’, with an overall accuracy of 0.86, suggesting results comparable to MFCC but a marginal reduction compared with Mel spectrograms.

EfficientNet, using Mel spectrograms, achieved a precision of 0.90 for ‘Bark’ and 0.73 for ‘Howl’, resulting in an overall accuracy of 0.81. Although EfficientNet exhibited balanced performance, its classification for the ‘Howl’ class was notably weaker. With MFCC features, EfficientNet obtained a precision and recall of 0.91 and 0.80 for ‘Bark’ and ‘Howl’, respectively, leading to an overall accuracy of 0.86. With LFCC features, EfficientNet achieved a precision of 0.92 for ‘Bark’ and 0.89 for ‘Howl’, resulting in an overall accuracy of 0.90, demonstrating higher efficacy with LFCCs.

The ResNet50 model, trained on Mel spectrograms, achieved a precision and recall of 0.91 and 0.80 for ‘Bark’ and ‘Howl’, respectively, with an overall accuracy of 0.86. Similar results were observed with MFCC features, achieving a precision and recall of 0.91 and 0.80 for both classes, and an overall accuracy of 0.86. LFCC features also provided a precision of 0.91 for ‘Bark’ and 0.80 for ‘Howl’, resulting in an overall accuracy of 0.86, indicating consistent performance across all feature types.

The ResNet152 model achieved a precision and recall of 0.90 and 0.73 for ‘Bark’ and ‘Howl’, respectively, with an overall accuracy of 0.81 when using Mel spectrograms, demonstrating slightly weaker performance for the ‘Howl’ class. With MFCC features, ResNet152 reached a precision and recall of 0.91 and 0.80, respectively, resulting in an overall accuracy of 0.86. LFCC features yielded a precision of 0.91 for ‘Bark’ and 0.80 for ‘Howl’, with an overall accuracy of 0.86, showing performance comparable to MFCC.

The comparative evaluation of various deep learning architectures (AlexNet, DenseNet, EfficientNet, ResNet50, and ResNet152) and feature extraction methods (Mel spectrograms, MFCCs, and LFCCs) for classifying dog vocalizations reveals significant variability in model performance based on feature selection. DenseNet exhibited the highest performance with Mel spectrograms, achieving 90% accuracy, while AlexNet also reached 90% accuracy with MFCCs. EfficientNet demonstrated notable success with LFCC features, achieving 90% accuracy. The ResNet models (ResNet50 and ResNet152) showed consistent performance across all feature extraction methods, achieving approximately 86% accuracy.

In addition to evaluating our proposed CNN-based architectures, we also compared their performance with traditional machine learning classifiers—namely Support Vector Machines (SVMs), K-Nearest Neighbors (KNNs), Random Forest, and Naive Bayes—using the same Mel, MFCC, and LFCC feature extraction techniques. As summarized in Table 1, these classical approaches achieved varying degrees of success:

- SVMs: exhibited consistent performance across all feature sets, with weighted average F1-scores around 0.81 (Mel), 0.86 (MFCCs), and 0.85 (LFCCs). Although robust, SVMs did not surpass the top-performing CNN configurations, which reached or exceeded 0.90 on some feature sets.
- KNNs: demonstrated comparatively weaker performance, with weighted average F1-scores of 0.64 (Mel) and around 0.76 (MFCCs, LFCCs). This method struggled to match the effectiveness of both the CNN-based models and the other classical approaches, indicating that KNNs may be less suited to the given data and feature distributions.
- Naive Bayes: achieved competitive results, particularly with MFCC and LFCC features, reaching a weighted average F1-score of 0.87. Although Naive Bayes approached the performance of some CNN models, it still fell short of the best CNN scores (e.g., 0.90+), suggesting that deep learning architectures better capture the complexity of the underlying representations.
- Random Forest: showed similar trends to Naive Bayes, achieving weighted average F1-scores around 0.86–0.87 with different feature sets. While Random Forest can leverage ensemble learning to yield strong results, it did not consistently outperform the strongest CNN configurations.

Overall, these comparisons indicate that while certain classical classifiers (notably Naive Bayes and Random Forest) provide respectable performance and can occasionally approach CNN-level metrics, they generally do not exceed the best results obtained by the proposed CNN architectures. The comparatively lower performance of KNNs further highlights the significance of model architecture and data representation in this task. By incorporating these traditional methods into our analysis, we have demonstrated that the improvements offered by the CNN-based approach are not merely incremental but represent a statistically significant advancement over more conventional classifiers. This comparison thus underscores the robustness and relevance of the proposed deep learning framework.

A general trend of reduced performance in classifying the ‘Howl’ class was observed, likely due to the complex acoustic properties of ‘Howl’ sounds and dataset imbalances. DenseNet and ResNet50 were identified as the most balanced models. These findings underscore the importance of selecting appropriate feature extraction methods and optimizing the pairing of deep learning architectures with features to enhance classification accuracy for dog vocalizations.

The experimental results presented in this section demonstrate the effectiveness of the proposed automatic sound detection and classification system in accurately identifying and classifying dog vocalizations. These results illustrate the performance of different convolutional neural network (CNN) models and feature extraction methods in achieving the research objectives.

Table 1 presents comprehensive performance metrics for each model and feature extraction method. Upon examination of the results, the impact of different feature extraction methods on the models is clearly evident. DenseNet achieved the highest success with Mel spectrograms (90%), EfficientNet with LFCCs (90%), and AlexNet with MFCCs (90%). This indicates that certain feature extraction methods may be more suitable for specific models. Among the models, ResNet50 stands out by demonstrating consistent performance across all feature extraction methods (86%). These results highlight the critical importance of not only model selection but also identifying the appropriate feature extraction method for the task of dog sound classification. Generally, ResNet and DenseNet architectures emerge as reliable options for such sound classification tasks due to their high and consistent performance.

EfficientNet shows the least tendency to overfit with both Mel spectrograms and MFCCs, indicating superior performance on unseen data. This reduced tendency to overfit can be attributed to the use of regularization techniques such as dropout and early stopping during model training. Additionally, EfficientNet’s architecture, which leverages compound scaling of depth, width, and resolution, significantly enhances the model’s generalization capabilities. DenseNet also performs close to EfficientNet with both audio representations. MFCCs are observed to be more effective in reducing the overfitting tendency of models compared with Mel spectrograms. The effectiveness of Mel-frequency cepstral coefficients (MFCCs) in the classification of barking and howling in dogs may be attributed to research indicating varying degrees of laryngeal lowering during these distinct vocalizations. This anatomical adaptability contributes to an expanded phonetic range that MFCCs can capture, thereby enhancing the accuracy of recognizing and differentiating between canine vocalizations, specifically barking and howling.

In this study, the effectiveness of different CNN architectures (AlexNet, DenseNet, EfficientNet, ResNet50, and ResNet152) and various feature extraction methods (Mel spectrograms, MFCCs, and LFCCs) on dog sound classification has been comprehensively examined. The findings reveal that model performances vary significantly depending on the feature extraction method used. The DenseNet architecture achieved the highest performance with an overall accuracy of 90% using Mel spectrograms, while AlexNet attained similar success with MFCC features, and EfficientNet reached the same accuracy level with LFCC features. The ResNet family (ResNet50 and ResNet152) exhibited consistent performance across all feature extraction methods, achieving accuracy rates of approximately 86%.

Notably, overall lower performance was observed in the “Howl” class compared with the “Bark” class; this can be attributed to the more complex acoustic features of howling sounds and potential imbalances within the dataset. These results indicate that the selection of deep learning architecture and feature extraction method significantly impacts classification performance in dog sound classification tasks, with each architecture performing better with specific feature extraction methods.

A general trend of reduced performance in classifying the ‘Howl’ class was observed, likely due to several factors. First, the ‘Howl’ class is underrepresented in the dataset, which results in fewer training instances and limits the model’s ability to learn robust features for this class. Second, howls may share temporal and spectral characteristics with other vocalizations, such as extended duration or overlapping frequency ranges, making them harder to distinguish. Additionally, compared with short and distinct vocalizations like barks, howls are acoustically more varied and continuous, which presents challenges for feature extraction and classification. These challenges emphasize the importance of balanced datasets and advanced feature engineering to improve performance in such complex sound classification tasks.

A key finding of this study is the distinct performance characteristics exhibited by linear-frequency cepstral coefficients (LFCCs) compared with other feature extraction methods. Unlike the logarithmic nature of the Mel scale, LFCCs’ linear filtering approach samples frequency bands at equal intervals, allowing for a more detailed representation of high-frequency components. This property enabled LFCCs, particularly with EfficientNet, to achieve superior classification performance, reaching an accuracy of 90% in distinguishing high-frequency components in canine vocalizations. DenseNet and ResNet architectures also demonstrated more consistent performance with LFCC features than with Mel spectrograms, suggesting that LFCCs’ linear filtering approach offers advantages in discerning sounds that span broad frequency ranges, such as barking and howling. These findings indicate that LFCCs’ linear frequency-based approach to feature extraction could be considered a viable alternative or complement to traditional Mel-based methods in specific auditory analysis tasks, such as dog sound classification.

A one-way Analysis of Variance (ANOVA) was performed to evaluate whether there were statistically significant differences in mean weighted average F1-scores among the nine models (AlexNet, DenseNet, EfficientNet, ResNet50, ResNet152, SVMs, KNNs, Naive Bayes, and Random Forest). The one-way ANOVA is a commonly used inferential statistical test for comparing the means of three or more independent groups, assessing whether observed variations reflect genuine effects rather than random fluctuations. In this study, each model’s performance was represented by three weighted average F1-scores obtained from distinct feature extraction methods (Mel, MFCCs, and LFCCs). Although this design introduces repeated measures at the feature level, the one-way ANOVA provides a preliminary understanding of variability in performance across the different models.

The one-way ANOVA yielded an F-value of approximately 5.05 (df = 8.18), which surpassed the critical F-value at the 0.05 significance level. Subsequent estimation of the *p*-value indicated that the result of *p* < 0.005 was well below the conventional 0.05 threshold. Consequently, the null hypothesis was rejected, indicating that there were statistically significant differences in the mean weighted average F1-scores among the nine models.

In summary, the experimental results validate the effectiveness of CNN models and feature extraction methods in behavioral analysis through sound. The findings underscore the importance of feature extraction in the classification of dog vocalizations. DenseNet and ResNet50 are established as highly effective models for classifying dog sounds, while EfficientNet demonstrates notable potential due to its robust generalization capabilities.

This study has a notable limitation: the dataset utilized consists solely of canine vocalizations sourced from a single context, with limited variability in breeds, environmental factors, and acoustic scenarios. Although the proposed models demonstrate strong performance on this dataset, their generalizability to broader and more diverse datasets remains uncertain. The lack of publicly available and diverse canine vocalization datasets restricts the range of conditions under which the models can be evaluated, potentially impacting external validity. To address this limitation, future research should prioritize the inclusion of larger and more varied datasets encompassing a broader spectrum of breeds, recording environments, and acoustic conditions. Such efforts would facilitate a more robust external validation of the proposed approach and enhance its applicability and reliability. Recent advances in the development and dissemination of comprehensive animal vocalization datasets provide a promising avenue to overcome these constraints. By systematically evaluating model performance across diverse datasets, this line of research can offer critical insights into the utility and reliability of deep learning-based classification methods for animal vocalizations, paving the way for further advancements in the field.

These findings make substantial contributions to the research objectives by demonstrating the viability and efficacy of deep learning models in the automatic detection and classification of dog vocalizations. The observed variations in performance across different models and feature extraction methods underscore the critical importance of judicious model selection and feature engineering to achieve high accuracy in audio classification tasks.

The insights gained from this study can be valuable for researchers and practitioners working on animal behavior analysis, veterinary sciences, and acoustic signal processing. Future work could explore the application of these models to a wider range of animal vocalizations or investigate the use of more advanced audio preprocessing techniques to further improve classification accuracy.

## 4. Conclusions

Animal vocalizations have been widely used by researchers for behavioral analysis. Accurate detection and classification of these vocalizations not only enhance the understanding of vocal patterns but also serve as a gateway to more detailed behavioral studies. By providing precise data on vocalizations, this system offers a tool to aid research in areas such as animal welfare, training, and interspecies communication.

This study presents an automatic sound detection and classification system for identifying animal vocalizations. The system employs convolutional neural network (CNN) models, which performed effectively across different spectrogram types. Specifically, AlexNet performed best with MFCCs, DenseNet with Mel spectrograms, and EfficientNet with LFCCs. Overfitting was mitigated, enhancing model performance. An automated sound analysis application was developed by integrating the classifier with an audio segmentation process.

Our investigation highlighted a deficiency in open-source audio datasets from controlled environments. Given the importance of canine vocalizations, more open-source datasets are needed. Although our datasets were limited to canine sounds, the classifier has potential for other species due to its learning-based nature.

The audio segmentation algorithm is suitable for controlled environments, but further work is needed for noisy, dynamic settings.

To adapt the system for real-world scenarios, which often involve noisy and dynamic environments, several potential approaches are suggested. Noise-reduction preprocessing techniques and environment-specific calibration can help mitigate the impact of environmental noise. Additionally, domain adaptation methods, such as transfer learning, can enable the model to generalize from controlled datasets to more diverse, real-world acoustic conditions. Data augmentation techniques simulating various environmental settings can further enhance the model’s robustness. Addressing these challenges will not only improve the system’s performance but also expand its applicability to tasks such as outdoor monitoring, animal shelters, or wildlife conservation research. The current classification is offline, but real-time implementation is feasible and should be tested.

The developed system bridges the gap between sound classification and behavior analysis, offering a foundational tool for researchers. By enabling the automatic classification of animal vocalizations, it supports studies in bioacoustic research and human sound analysis, opening avenues for further exploration in this interdisciplinary field.

## Figures and Tables

**Table 1:** Experimental results.

		Bark				Howl					Macro Avg			Weighted Avg		
Model	Feature	Precision	Recall	F1-Score	Support	Precision	Recall	F1-Score	Support	Accuracy	Precision	Recall	F1-Score	Precision	Recall	F1-Score
AlexNet	Mel Spectrogram	0.83	0.83	0.83	12	0.78	0.78	0.78	9	0.81	0.81	0.81	0.81	0.81	0.81	0.81
AlexNet	MFCC	0.92	0.92	0.92	12	0.89	0.89	0.89	9	0.90	0.90	0.90	0.90	0.90	0.90	0.90
AlexNet	LFCC	0.90	0.75	0.82	12	0.73	0.89	0.80	9	0.81	0.81	0.82	0.81	0.83	0.81	0.81
DenseNet	Mel Spectrogram	0.92	0.92	0.92	12	0.89	0.89	0.89	9	0.90	0.90	0.90	0.90	0.90	0.90	0.90
DenseNet	MFCC	0.91	0.83	0.87	12	0.89	0.89	0.89	9	0.86	0.85	0.86	0.86	0.86	0.86	0.86
DenseNet	LFCC	0.91	0.83	0.87	12	0.80	0.89	0.84	9	0.86	0.85	0.86	0.86	0.86	0.86	0.86
EfficientNet	Mel Spectrogram	0.90	0.75	0.82	12	0.73	0.89	0.80	9	0.81	0.82	0.81	0.81	0.83	0.81	0.81
EfficientNet	MFCC	0.91	0.83	0.87	12	0.80	0.89	0.84	9	0.86	0.85	0.86	0.86	0.86	0.86	0.86
EfficientNet	LFCC	0.92	0.92	0.92	12	0.89	0.89	0.89	9	0.90	0.90	0.90	0.90	0.90	0.90	0.90
ResNet50	Mel Spectrogram	0.91	0.83	0.87	12	0.80	0.89	0.84	9	0.85	0.86	0.86	0.86	0.86	0.86	0.86
ResNet50	MFCC	0.91	0.83	0.87	12	0.80	0.89	0.84	9	0.86	0.85	0.86	0.86	0.86	0.86	0.86
ResNet50	LFCC	0.91	0.83	0.87	12	0.80	0.89	0.84	9	0.86	0.85	0.86	0.86	0.86	0.86	0.86
ResNet152	Mel Spectrogram	0.90	0.75	0.82	12	0.73	0.89	0.80	9	0.81	0.81	0.82	0.81	0.83	0.81	0.81
ResNet152	MFCC	0.91	0.83	0.87	12	0.80	0.89	0.84	9	0.86	0.85	0.86	0.86	0.86	0.86	0.86
ResNet152	LFCC	0.91	0.83	0.87	12	0.80	0.89	0.84	9	0.86	0.85	0.86	0.86	0.86	0.86	0.86
SVM	Mel Spectrogram	0.83	0.83	0.83	12	0.78	0.78	0.78	9	0.81	0.81	0.81	0.81	0.81	0.81	0.81
SVM	MFCC	0.91	0.83	0.87	12	0.80	0.89	0.84	9	0.86	0.85	0.86	0.86	0.86	0.86	0.86
SVM	LFCC	0.90	0.83	0.87	12	0.80	0.85	0.82	9	0.85	0.85	0.84	0.85	0.85	0.85	0.85
KNN	Mel Spectrogram	0.70	0.70	0.70	12	0.50	0.63	0.56	9	0.64	0.60	0.67	0.63	0.64	0.64	0.64
KNN	MFCC	0.81	0.80	0.80	12	0.70	0.70	0.70	9	0.76	0.76	0.75	0.75	0.76	0.76	0.76
KNN	LFCC	0.81	0.80	0.80	12	0.70	0.70	0.70	9	0.76	0.76	0.75	0.75	0.76	0.76	0.76
Naive Bayes	Mel Spectrogram	0.91	0.83	0.87	12	0.80	0.89	0.84	9	0.86	0.85	0.86	0.86	0.86	0.86	0.86
Naive Bayes	MFCC	0.90	0.83	0.86	12	0.78	0.89	0.84	9	0.87	0.84	0.86	0.85	0.87	0.87	0.87
Naive Bayes	LFCC	0.90	0.83	0.86	12	0.78	0.89	0.84	9	0.87	0.84	0.86	0.85	0.87	0.87	0.87
Random Forest	Mel Spectrogram	0.91	0.83	0.87	12	0.80	0.89	0.84	9	0.86	0.85	0.86	0.86	0.86	0.86	0.86
Random Forest	MFCC	0.90	0.83	0.86	12	0.78	0.89	0.84	9	0.87	0.84	0.86	0.85	0.87	0.87	0.87
Random Forest	LFCC	0.91	0.83	0.87	12	0.80	0.89	0.84	9	0.86	0.85	0.86	0.86	0.86	0.86	0.86

## Data Availability

The data presented in this study are openly available in https://github.com/mkaraaslan-dev/fully-automatic-voice-analysis-system (accessed on 11 December 2024).
